# A QM-MD simulation approach to the analysis of FRET processes in (bio)molecular systems. A case study: complexes of *E. coli* purine nucleoside phosphorylase and its mutants with formycin A

**DOI:** 10.1007/s00894-015-2602-8

**Published:** 2015-03-10

**Authors:** M. Sobieraj, K. A. Krzyśko, A. Jarmuła, M. W. Kalinowski, B. Lesyng, M. Prokopowicz, J. Cieśla, A. Gojdź, B. Kierdaszuk

**Affiliations:** 1 Department of Biophysics, Institute of Experimental Physics, Faculty of Physics, University of Warsaw, 02-089 Warsaw, Poland; 2 Bioinformatics Laboratory, Medical Research Centre, Polish Academy of Sciences, 02-106 Warsaw, Poland; 3 Department of Biochemistry, Nencki Institute of Experimental Biology, Polish Academy of Sciences, 02-093 Warsaw, Poland; 4 Department of Technology and Biotechnology, Faculty of Chemistry, Warsaw University of Technology, 00-664 Warsaw, Poland

**Keywords:** Deprotonation, Emission spectroscopy, Excited state, Formycin A, FRET, PNP, QC-MD, SCF-CI, Simulations, Tyrosinate anion

## Abstract

Predicting FRET pathways in proteins using computer simulation techniques is very important for reliable interpretation of experimental data. A novel and relatively simple methodology has been developed and applied to purine nucleoside phosphorylase (PNP) complexed with a fluorescent ligand — formycin A (FA). FRET occurs between an excited Tyr residue (D*) and FA (A). This study aims to interpret experimental data that, among others, suggests the absence of FRET for the PNPF159A mutant in complex with FA, based on novel theoretical methodology. MD simulations for the protein molecule containing D*, and complexed with A, are carried out. Interactions of D* with its molecular environment are accounted by including changes of the ESP charges in S_1_, compared to S_0_, and computed at the SCF-CI level. FRET probability *W*_*F*_ depends on the inverse six-power of the D*-A distance, *R*_*da*_. The orientational factor 0 < k^2^ < 4 between D* and A is computed and included in the analysis. Finally *W*_*F*_ is time-averaged over the MD trajectories resulting in its mean value. The red-shift of the tyrosinate anion emission and thus lack of spectral overlap integral and thermal energy dissipation are the reasons for the FRET absence in the studied mutants at pH 7 and above. The presence of the tyrosinate anion results in a competitive energy dissipation channel and red-shifted emission, thus in consequence in the absence of FRET. These studies also indicate an important role of the phenyl ring of Phe159 for FRET in the wild-type PNP, which does not exist in the Ala159 mutant, and for the effective association of PNP with FA. In a more general context, our observations point out very interesting and biologically important properties of the tyrosine residue in its excited state, which may undergo spontaneous deprotonation in the biomolecular systems, resulting further in unexpected physical and/or biological phenomena. Until now, this observation has not been widely discussed in the literature.

## Introduction

**Fig. 1:**
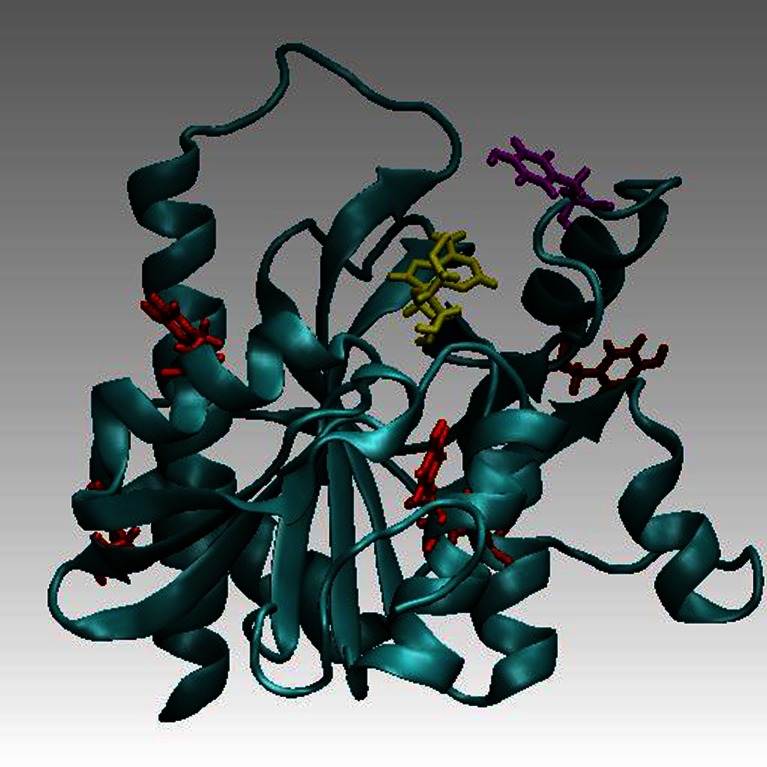
*E. coli* PNP (*ribbon representation*) complexed with FA (*yellow*). The closest to FA tyrosine residue (Tyr160) is in magenta. Other tyrosines: Tyr27, Tyr52, Tyr72, Tyr186, and Tyr173 (going counterclockwise, when starting from the upper, left corner) are in *red*

Nonradiative Förster resonance energy transfer occurs from a donor (D) molecule to an acceptor (A) molecule, D* + A → D + A*, and results from a dipole-dipole interaction between their electronic states. Transfer occurs when the oscillations of an optically induced electronic coherence on D* are resonant with the energy gap of A. FRET is a phenomenon used in molecular biophysical studies, including protein conformational changes, protein-protein interactions or protein–DNA interactions, as well as in fluorescence microscopy applications. Predicting FRET pathways in proteins using computer aided simulation techniques is very important for the reliable interpretation of experimental data. In this study, a novel and relatively simple methodology has been developed and applied to purine nucleoside phosphorylase (PNP) from *E. coli* complexed with a fluorescent ligand — formycin A (FA). Purine nucleoside phosphorylases (PNPs, E.C. 2.4.2.1) use orthophosphate (P_i_) to cleave the N-glycosidic bond of β-(deoxy) ribonucleosides to yield α-(deoxy) ribose 1-phosphate and the free purine base. *Escherichia coli* PNP, the product of the *deoD* gene, which cleaves Ado more effectively than Ino and Guo, has been reported to migrate as a hexamer. This is in contrast to the trimeric PNPs, which accept xanthosine (Xao) with comparable efficiency to Guo and Ino, the usual physiological substrates for trimeric PNPs. Absorption and emission spectra of *E. coli* PNP result from the presence of tyrosine residues and are characterized by maxima around 277 and 305 nm, respectively. Since FA exhibit absorption and emission spectra red-shifted relative to PNP, with the maxima at 295 and 340 nm, respectively, one observes the existence of fluorescence resonance energy transfer (FRET) processes between an excited Tyr residue (D*) of PNP and FA (A) [1]. A ribbon model of *E. coli* PNP, with highlighted tyrosines and FA is presented in Fig. 1 (below):

Apart from the wild-type PNP containing Phe159 and Tyr160, three mutants have been cloned, extracted, purified, and studied with molecular spectroscopy methods. These are described in Table 1.

**Table 1:** *E. coli* purine nucleoside phosphorylase (PNP), the product of the *deoD* gene, and its mutants

Case	Description of the enzyme molecules	Denotations with one letter aminoacids coding
1	Wild type PNP (with Phe159 – Tyr160)	PNP
2	Tyr160 → Ser160	PNP Y160S
3	Phe159 → Ala159	PNP F159A
4	Phe159 → Tyr159	PNP F159Y

Whereas the wild-type PNP exhibit the FRET phenomenon, it is not observed for the above mentioned mutants. In particular, since FRET is not observed for mutant number 2, Tyr160 in its S_1_ excited state is identified as the most probable energy donor, and FA as the acceptor [1].

## Experimental materials and methods

### Formycin A and *E. coli* PNP wild type

Formycin A (FA), N-2-hydroxyethyl-piperazine-N’-2-ethanesulfonic acid (Hepes), were products of Sigma Chemical Co. (St. Louis, MO, USA). All solutions were prepared with high-quality MilliQ water. Reagents were of the highest commercially available quality, and only those of spectral grade, checked by UV absorption and/or fluorescence emission, were employed.

The concentration of FA was determined spectrophotometrically at pH 7.0, λ_max_ 294 nm (ε 10.3 × 10^3^ M^−1^ cm^−1^). Measurements of pH (+/−0.05) were with a Jenway (UK) pH-meter equipped with a combination semi-micro electrode and temperature sensor.

Cloned PNP from *E. coli*, the product of the *deoD* gene, was purified to apparent homogeneity and final specific activity about 100 U mg^−1^ [2]. The enzyme consists of six identical subunits, and enzyme concentrations are expressed in terms of the native hexamer (λ_max_ 277, ε 43.2 × 10^3^ M^−1^ cm^−1^), unless otherwise stated. The molar extinction coefficient for the native hexamer ε 43200 M^−1^ cm^−1^ was determined [1] and a molecular mass of 159 kDa calculated from the amino acid sequence [3]. Enzyme activity was monitored at 25 °C in 50 mM phosphate buffer (pH 7.0), spectrophotometrically by the coupled xanthine oxidase procedure with Ino as a substrate [4,5], and/or by following the changes in absorption of m^7^Guo at 260 nm as the substrate [6].

### PNP mutants

#### Plasmid constructs

The *E. coli* strain with pSE380 plasmid carrying the *E. coli* PNP DeoD gene was a kind gift from Dr. Joanne L. Turnbull. The plasmid was a template in site-directed mutagenesis reaction performed with the use of Invitrogen GeneTailor^TM^ Site-Directed Mutagenesis System (Invitrogen) according to the manufacturer’s protocol. Forward primers used for mutagenesis were designed as follows (changed TTC phenyloalanine codon underlined): 5’-ACCTGTTCTCCGCTGACCTGTACTACTCTCCGG-3’ (for F159Y mutation) and 5’-TGTTCTCCGCTGACCTGGCCTACTCTCCGG-3’ (for F159A mutation). The reverse primer was the same for both reactions: 5’-CAGGTCAGCGGAGAACAGGTTACCCACGCG-3’. The synthesis of oligonucleotide primers and sequencing of the mutated PNP gene were done by Genomed S.A.

#### Overexpression of mutated PNP

*E. coli* DH5α^TM^-T1® cells containing pSE380 plasmid with the PNP F159Y or PNPF159A gene were grown in a Superbroth medium (2 % peptone, 0.2 % sodium phosphate (dibasic), 0.1 % potasium phosphate (monobasic), 0.8 % sodium chloride, 1.5 % yeast extract, 0.2 % glucose, and 50 μg/ml ampicillin) at 37 °C with constant shaking (250 rpm) to OD_600_ of 0.8 before inducing the protein overproduction with 1 mM isopropyl α-D-1-thiogalactopyranoside (IPTG). The cells were grown for an additional 4 h in the same conditions and harvested by centrifugation for 20 min at 4000 × g at 4 °C.

### Purification of F159Y

The cell pellet, obtained from 6 l of bacterial culture, was resuspended in 100 ml of homogenizing buffer H (100 mM Tris–HCl, pH 7.6, 2 mM EDTA, 10 % glycerol (v/v), 1 mM DTT, 50 mM NaCl, 0.4 mg/ml lysozyme, and 0.015 mg/ml PMSF), incubated on ice for 30 min, sonicated and centrifuged for 20 min at 43,000 × g at 4 °C. The supernatant was filtered through four layers of gauze, supplemented with an equal volume of 100 mM Tris–HCl buffer (pH 7.6) containing 2 mM EDTA, 10 % glycerol (v/v), and 1 mM DTT (buffer A) and applied onto Q-Sepharose^TM^ Fast Flow column (120 ml, GE Healthcare). The column was washed with 1.2 l of buffer A and the enzyme was eluted with a 1.2 l linear gradient of 0.05–0.3 M KCl in buffer A. Fractions were assessed for PNP activity (spectrophotometric assay) and purity (SDS-PAGE). The purest fractions were pooled (235 ml), concentrated in Amicon stirred cell (Millipore) to 56 ml and supplemented with an equal volume of 2 M ammonium sulfate (SA). The enzyme was applied onto Phenyl-Sepharose CL-4B (30 ml, GE Healthcare) equilibrated with 50 mM Hepes pH 7.6 containing 1 M SA. The column was washed with 1 l of the same buffer and PNP was eluted with a 1 l linear gradient of 1–0 M SA in 50 mM Hepes pH 7.6. The purest fractions were pooled (224 ml), concentrated in Amicon stirred cell to 14 ml, and dialyzed against 10 mM Hepes pH 7.0 with two changes of buffer. The enzyme was applied onto DEAE-cellulose (20 ml, Whatman) washed with 250 ml of 50 mM Hepes pH 7.0 and then, with 100 ml of 100 mM Hepes pH 7.0. PNP F159Y, the protein was eluted with 800 ml linear gradient of 0–0.3 M NaCl in buffer A. The fractions containing PNP protein were pooled (150 ml), concentrated in Amicon stirred cell and dialized against two changes of 50 mM Hepes pH 7.0. The final PNP preparation (7.5 ml) contained 32 mg of protein per ml and was over 98 % homogenous. The protein was divided into small aliquots and kept frozen at −20 °C until further use.

### Purification of F159A

The enzyme was purified using FPLC ÄKTApurifier 10 system (GE Pharmacia). The 2 l bacterial culture was divided into 500 ml aliquots, centrifuged and pellets were frozen at −20 °C. The pellet of cells corresponding to 500 ml of bacterial culture was resuspended in 10 ml of buffer H, incubated on ice for 30 min, sonicated and centrifuged for 20 min at 43,000 × g at 4 °C. The supernatant was supplemented with an equal volume of buffer A passed through MF Millipore membrane mix cellulose esters hydrophilic 0.45 µm filter and applied onto HiLoad 16/10 Q-Sepharose HP column (20 ml) equilibrated with buffer A. The column was washed with 60 ml of buffer A followed by 60 ml of buffer A containing 150 mM KCl. The enzyme was eluted with a 280 ml linear gradient of 150–250 mM KCl in buffer A. The purest fractions, assessed by SDS-PAGE, were pooled and precipitated with SA (0–80 % saturation). The protein pellet obtained by centrifugation at 47000 × g for 20 min at 4 °C was frozen at −20 °C. The above procedure was performed for each batch of bacterial pellet. The resulting protein pellet, corresponding to 2 l of bacterial culture, was divided into two portions and subjected to further purification, one portion at a time. The pellet was resuspended in 50 mM Tris–HCl pH 7.6 containing 1 M SA (buffer B), filtered as described above and loaded onto HiPrep 16/10 Phenyl-Sepharose column (20 ml resin). The column was washed with 40 ml of buffer B and eluted with a 320 ml linear gradient of 1–0 M SA in buffer B. The purest fractions were pooled and precipitated with 0–80 % saturation of SA. The protein pellet was resuspended in 1 ml of 50 mM Hepes pH 7.0 and dialyzed against three changes of the same buffer (1 l for 1 h, 1 l for 3 h and 2 l overnight). The final 2.9 ml of homogenous PNPF159A preparation contained 31.6 mg of protein. The protein was divided into small aliquots and kept frozen at −20 °C until further use.

### Steady-state absorption and fluorescence measurements

Ultraviolet absorption was monitored with a Varian (Australia) Cary 50 recording instrument, fitted with a thermostatically-controlled cell compartment at 25 °C, using 5-mm pathlength cuvettes. Steady-state fluorescence emission and excitation spectra were measured with a Spex (USA) FluoroMax spectrofluorimeter at 25 °C, set up in the single photon counting mode, with 4-nm spectral resolution for excitation and emission. Samples were prepared in 500–570 μl of 50 mM Hepes (pH 7, pH 8.3), phosphate buffer (pH 7, pH 8.3), and acetate buffer (pH 4, pH 5, pH 6) in 5×5-mm Suprasil cuvettes. Fluorescence emission (λ_ex_ 280, 295, and 313 nm) and excitation (λ_em_ 305, 340, and 365 nm) spectra of PNP, FA, and their mixtures were recorded. Background emission (~0.5 %), was eliminated by subtracting the signal for the buffer.

## Theoretical methods and simulations

### Molecular mechanics (MM) and dynamics (MD) simulations

MD simulations were carried out in the canonical ensemble for the wild-type *E. coli* PNP, complexed with FA, as well as for its two mutants with the following mutations: Phe159- > Ala159 and Phe159- > Tyr159, denoted PNPF159A and PNPF159Y, respectively. NAMD/VMD (http://www.ks.uiuc.edu/Research/namd/) simulation MM and MD environment was used. CHARMM force field was applied (http://www.charmm.org/). Simulations accounted also for S_1_ excited states of Tyr160* (in PNP), Tyr159* (in PNPF159Y), as well as FA* in PNP. The assumption was for only one excited residue in each case under the study. This, in particular, meant that either Tyr160 or Tyr159 were excited. In addition, the deprotonated form of Tyr 160 (Tyr160^(−)^) was considered in its ground as well as excited states. All MD simulations were carried out in periodic boundary conditions, with a box of the size 49×78×84 Å, containing the enzyme molecule surrounded by about 10*10^3^ water molecules and with ionic strength of 0.05 mM model salt. MD simulations were proceeded by careful energy minimization with the norm of the energy gradient below 10^−2^ kcal mol^-1^ Å^-1^. The crystallographic structure of *E. coli* PNP in complex with FA from Protein Data Bank (PDB code: 1PR1) was used for the modeling studies.

### Quantum mechanical (QM) calculations

QM computations for Tyr and FA in their ground S_0_ as well as in excited S_1_ states were carried out using the TURBOMOLE 6.1 package (http://www.turbomole.com/) at the SCF-CI level. TZVP split valence, triple zeta basis sets was used (http://www.gaussian.com/g_tech/g_ur/m_basis_sets.htm). Deprotonated Tyr^(−)^ in its ground and excited states was also considered. In particular, the ESP charges were computed for all residues/molecules. Changes in the interaction potentials in S_1_ in comparison to S_0_ were accounted for by modifying the atomic charges. In the ground state, the CHARMM charges were applied. In the excited state, the CHARMM charges were modified by the changes of the quantum ESP charges when coming from the ground to the excited state. In this way the original CHARMM parameterization was applied including relative changes in the electrostatic interactions generated by the quantum-mechanical ESP charges when coming from the ground to the excited state or from the neutral to the deprotonated forms.

### Virtual titration

In order to define initial conditions for MM and MD simulations at the microscopic level, the presence or absence of all protons at dissociable groups has to be defined. Typically, one assumes standard pK values for all aminoacids, and their total charges are determined, further resulting in well-defined atomic charges. In the present study, we discovered that the FRET phenomenon is sensitive to proton ionization states of residues in PNP, in particular in the area of the FA binding site. Therefore, PNP and its mutants in complex with FA were virtually titrated for pH in the range of 4.0–11.0. The Poisson-Boltzmann formalism along with a Monte-Carlo procedure was used to determine mean electrostatic energies of protons in their binding-sites. The above-mentioned formalisms and numerical methodologies are presented, in particular, in [7,8].

### Simulations of FRET processes

MD simulations for the protein molecule containing D* (here Tyr160*) and complexed with A (here formycin A) were carried out. Interactions of D* with its molecular environment are accounted for by including changes of the ESP charges in S_1_, compared to S_0_, and computed at the SCF-CI level. *W*_*F*_, frequency of FRETs per ns, depends on the inverse six-power of the D*-A distance, *R*_*da*_.

1 $$ {W}_F(t)=\frac{3{k}^2(t)}{2{\tau}_d}{\left(\frac{R_F}{R_{d\;a}\;(t)}\right)}^6 $$

where *τ*_*d*_ = 2.57 ns is the experimental mean life-time of the donor. Förster radius *R*_*F*_ is given by the following formula,

2 $$ {R}_F=0.2108{\left({n}^{-4}{Q}_D\left\langle {k}^2\right\rangle J\right)}^{\frac{1}{6}} $$

See *Förster radius for tyrosine and aromatic ligand* for its estimate. *R*_*F*_ in [Å]. *Q*_*D*_ is the fluorescence quantum yield of the donor in the absence of the acceptor, *κ* is the dipole orientation factor, *n* is the refractive index of the medium, *J* is the spectral overlap integral:

3 $$ J={\displaystyle \underset{0}{\overset{\infty }{\int }}{f}_D\left(\lambda \right){\varepsilon}_A}\left(\lambda \right)\;{\lambda}^4d\lambda $$

where *f*_*D*_ is the normalized fluorescence spectrum of D, *ε*_*A*_ is the molar excitation coefficient of the A, λ is the wavelength in [nm].

The orientation factor 0 < k^2^ < 4 between D* and A is computed and included in the analysis,

4 $$ k={\overrightarrow{\upmu}}_d\;{\overrightarrow{\upmu}}_a-3\left(\frac{{\overrightarrow{R}}_{da}}{R_{da}}\;{\overrightarrow{\upmu}}_d\right)\;\left(\frac{{\overrightarrow{R}}_{da}}{R_{da}}{\overrightarrow{\upmu}}_a\right) $$

μ_d_ and μ_a_ are normalized electric transition moments of the donor and acceptor, respectively. In the course of MD they can change their orientations. *R*_*da*_ is vector directed from D* to A. A few definitions of this vector have been considered, however, because final results did not depend much on the applied definition, a conventional, “spectroscopic definition” was applied. *R*_*da*_ was attached to the geometric center of the Tyr ring and directed to the mid point of the bond linking the six and five membered rings of FA.

The above quantities are time-averaged over the MD trajectories resulting in their time-averaged mean values,

5 $$ \left\langle {k}^2\right\rangle =\frac{1}{T}{\displaystyle \underset{0}{\overset{T}{\int }}{k}^2(t)\;dt} $$

6 $$ \left\langle {W}_F\right\rangle =\frac{1}{T}{\displaystyle \underset{0}{\overset{T}{\int }}{W}_F(t)\;dt} $$

One assumes ergodic hypothesis which states that the time-averages are equal to ensemble averages, measured experimentally. The quantum yield of the energy transfer transition is given by:

7 $$ E\left({R}_{da}(t), {k}^2(t)\right)=\frac{1}{1+{\left({w}_F(t){\tau}_d\right)}^{-1}} $$

and the mean quantum yield of the energy transfer is:

8 $$ \left\langle E\right\rangle =\frac{1}{T}{\displaystyle \underset{0}{\overset{T}{\int }}\frac{1}{1+{\left({W}_F(t){\tau}_d\right)}^{-1}}\;dt} $$

Other possible deactivation paths, including thermal deactivation to the molecular environment, in particular water solvent, were also considered.

Similar methodology, using a combination of MC and MD simulations, has been applied to a model molecular system — polyproline with two dyes attached to its ends ([9]; [10]). However, according to our knowledge, there are no studies based on first principles FRET models applied to such complex systems like PNP and its mutants, including protonation-deprotonation phenomena in the excited state of D* (see below).

## Results and discussion

### Fluorescence properties of *E. coli* PNP and ligand (inhibitor)

**Fig. 2:**
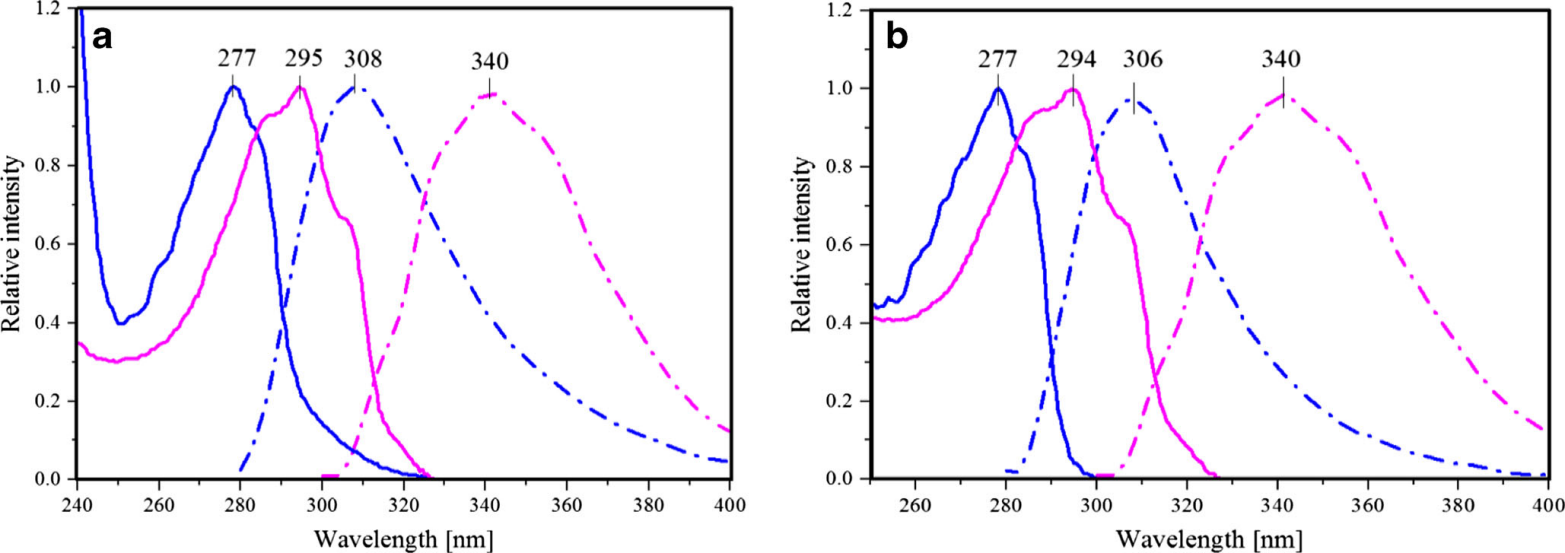
UV absorption (*solid*) and fluorescence emission (*dash-dot*) spectra of *E. coli* PNP mutatnts PNPF159Y, PNPF159A, and FA (*magenta*) in 50mM Hepes, pH 7: **a** PNPF159Y (*blue*), and neutral form of FA (*magenta*); **b** PNPF159A (*blue*), and neutral form of FA (*magenta*). Maximum intesities of the absorption and emission spectra of PNP (λ_ex_ 270 nm) and FA (λ_ex_ 295 nm) are normalized to unity

**Fig. 3:**
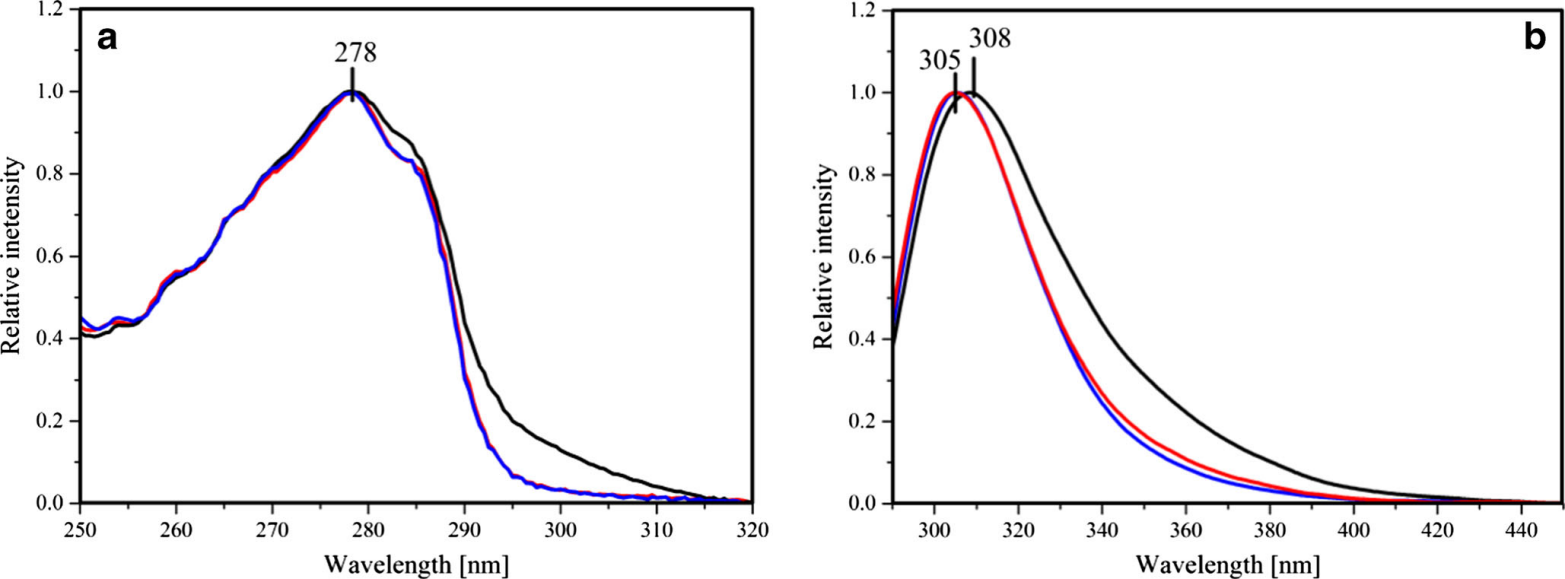
Absorption **a** and emission after excitation at 280 nm **b** normalized spectra of wild type PNP (*red*), PNPF159Y (*black*), and PNPF159A (*blue*)

The maxima of the absorption and emission spectra of PNPF159Y are located at 278 and 308 nm, respectively (Fig. 2a), typical for proteins containing tyrosine, but no tryptophan [11], consistent with the fact that each of the subunits of that PNP mutant contains seven tyrosine residues and no tryptophan [12]. However, the fluorescence maximum is slightly shifted to higher wavelengths compared to wild type PNP [13], accompanied by higher values of the half width of emission band and long-wavelength extended absorption spectrum of PNPF159Y (Figs. 2a and 3). A probable explanation of mentioned behavior might be the presence of the tyrosinate anion, which exhibits excitation maximum at 295 nm and emission shifted to long wavelengths (352 nm in solution) [14]. Indications for the presence of the tyrosinate anion in PNPF159Y are visualized by absorbance and emission spectra of the wild type and both mutants (Fig. 3), where aforementioned characteristic features are present. On the contrary, emission maximum of PNPF159A is at 306 nm, which is close to the wild type PNP (Fig. 2b). By contrast, the nucleoside ligand FA, specific inhibitor of the bacterial enzyme [15,16], exhibits absorption and emission spectra red-shifted relative to wt PNP and its mutants, with the maxima at 295 and 340 nm, respectively (Fig. 2).

This permits selective excitation of FA in the enzyme-ligand complexes at excitation wavelengths above 295 nm (where tyrosine residues do not absorb). Selective observation of PNP fluorescence is possible in the range 290–310 nm, and fluorescence of ligand (FA) at wavelengths above 360 nm, where the contribution of protein fluorescence is usually negligible.

### Förster radius for tyrosine and aromatic ligand

The existence of fluorescence resonance energy transfer (FRET) between protein tyrosine residues and a bound aromatic ligand should be consistent with the good alignment of the respective donor (tyrosine) emission and acceptor (FA) absorption spectral bands. The Förster radius was calculated as described elsewhere for other analogous systems [17], for FRET from Tyr residues to m^1^FA [1], which does not exhibit tautomerism. This gave *R*_*F*_ = 24 Å, comparable to values for similar systems, and to the dimensions of the enzyme subunit in the crystal [18]. Since the wt enzyme contains six tyrosine residues per subunit, at least several of these should be located within range of the Förster distance to the acceptor.

### Absence of FRET between protein tyrosine residue(s) and the bound FA

Fluorescence emission of both PNPF159Y and PNPF159A mutants overlaps with the absorption of FA ligand, in the extent convenient for FRET incident (Fig. 2a and b). For both mutants, the overlap integral is sufficient for energy transfer occurrence, which should result in enhanced FA emission in the complexes [19].

**Fig. 4:**
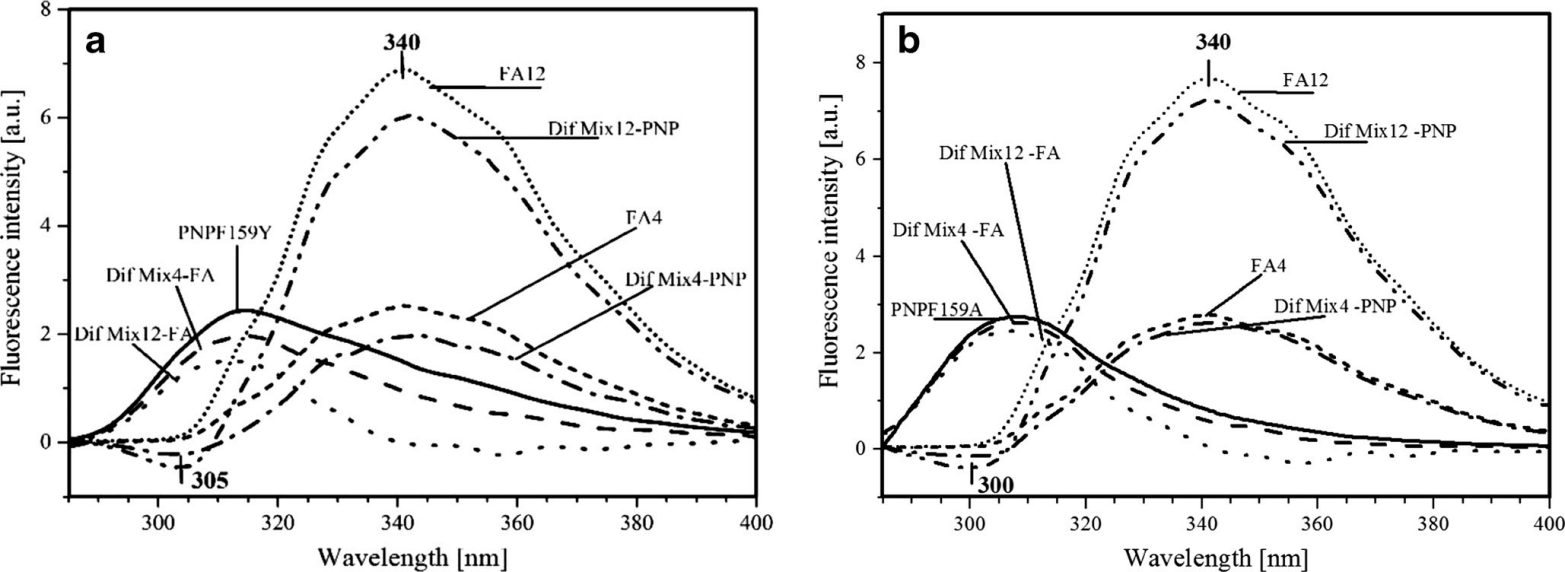
Fluorescence emission (λex 280 nm) spectra of PNPF159Y (**a**), PNPF159A (**b**), 4 μM (FA4), and 12 μM (FA12) FA in 50mM Hepes buffer (pH 7), and fluorescence-emission difference (λex 280 nm) spectra after subtraction of FA at both concentrations (Dif Mix4-FA, Dif Mix12-FA), and after substraction of PNPF159Y (**a**), or PNPF159A **b** at both concentrations of FA (Dif Mix4-PNP, Dif Mix12-PNP)

**Fig. 5:**
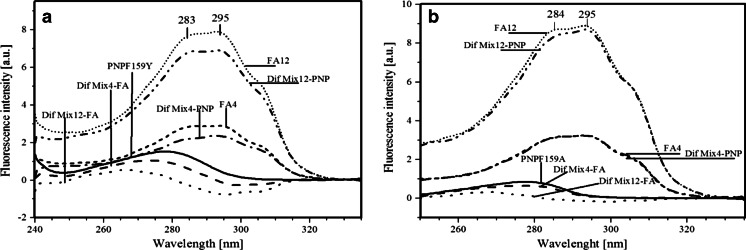
Fluorescence excitation (λem 340 nm) spectra of PNPF159Y (**a**), PNPF159A (**b**), 4 μM (FA4), and 12 μM (FA12) FA in 50mM Hepes buffer (pH 7), and fluorescence-excitation difference (λem 340 nm) spectra after subtraction of FA at both concentrations (Dif Mix4-FA, Dif Mix12-FA), and after substraction of PNPF159Y (**a**), or PNPF159A **b** at both concentrations of FA (Dif Mix4-PNP, Dif Mix12-PNP)

However, emission (Fig. 4) and excitation (Fig. 5) fluorescence spectra show that in both mutants, instead of FRET the protein fluorescence quenching by FA is observed, without affecting the location of the characteristic fluorescence maxima in proteins. This means that no changes in properties of aminoacid residues were caused by ligand binding (see difference-emission and difference-excitation spectra after FA subtraction).

However, as we can see in Figs. 4 and 5, the extent of enzyme quenching by FA depends on the type of mutant. The PNPF159Y fluorescence is much more sensitive for the FA presence, whereas PNF159A quenching is at least three times weaker than tyrosine mutant.

In addition, we can also observe quenching of ligands fluorescence (difference spectra after PNP substraction) that is caused by the protein mutants, and escalated with an increasing concentration of aforementioned, possibly due to extended interactions with the enzymes by creating complexes. As previously, this quenching depends on the type of mutant. Additionally, in Fig. 4 we observe blue-shift of the fluorescence minimum of both mutants. Probably, the reason is subtraction of ligands spectra, that are also quenched by interaction with the enzyme. This indicate the absence of the energy transfer between protein mutants and ligand, in contrast with considerations based on FRET in the wt PNP-FA complex, and supported by the difference fluorescence emission spectra of the mixture of wt PNP and FA. The latter clearly shows enhancement of FA fluorescence spectra due to the energy transfer from Tyr residues [1].

It is worth noting that the magnitude of quenching of FA fluorescence depends on the type of mutant. Both figures Figs. 4 and 5, demonstrates that decrease of FA emission is noticeably larger in the presence of PNPF159Y mutant than that of PNPF159A (Fig. 5a and Fig. B), respectively. The reason of aforementioned situation might be the presence of additional hydroxyl group in Tyr-159, which might change for example the transition moment or spatial arrangement of ligand and aminoacid residues in the active site, which could prevent energy transfer. In the case of PNPF159A mutant, the possible lack of energy transfer might be due to the absence of phenyl ring, the major difference between the wild type PNP and the PNPF159Y mutant. This could indicate an important role of the phenyl ring of Phe-159 in the energy transfer and in the binding of FA, which is supported by the high dissociation constant (K_d_) and thus the weak association of FA to PNPF159A. Additionally, we also observe concentration-dependent quenching of the FA fluorescence by both mutants, which is also in line with the absence of FRET.

**Fig. 6:**
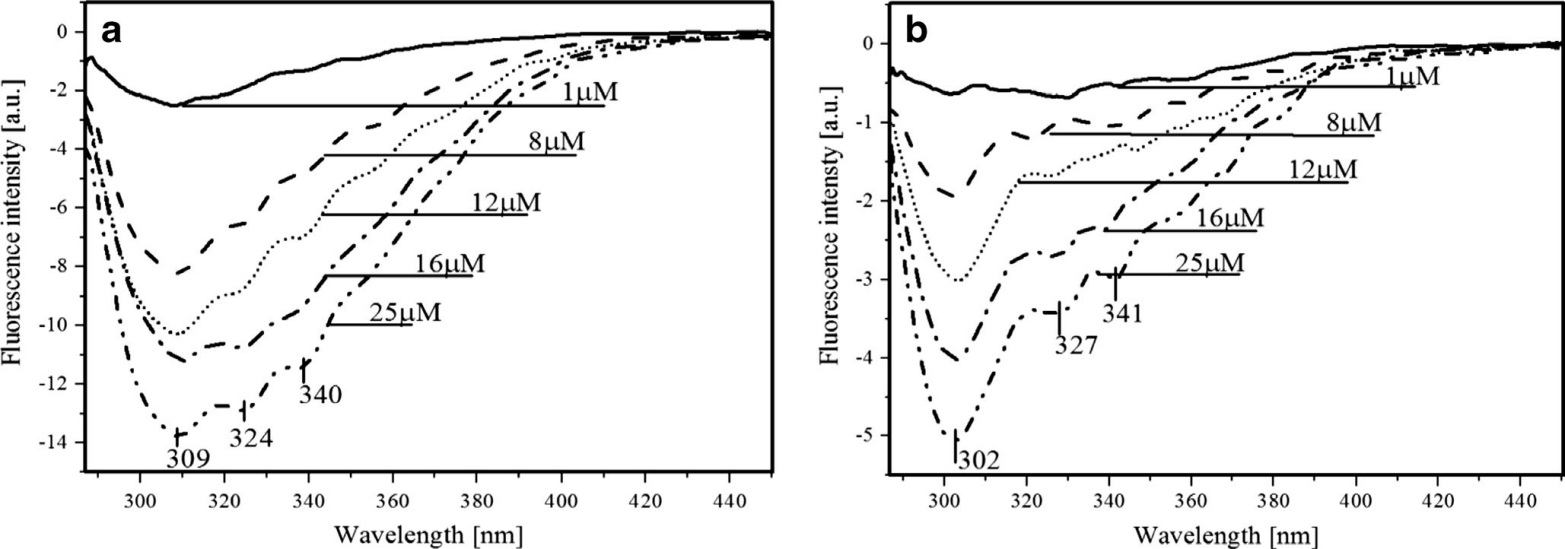
Fluorescence-emission difference (λ_ex_ 280 nm) spectra after subtraction of different FA concentrations and PNPF159Y **a** or PNPF159A **b** in 50mM Hepes pH 7

Fluorescence-emission difference spectra after subtraction of both components (Fig. 6a and b) confirm quenching of the enzymes and ligand in both types of complexes that enhance with increasing ligand concentration. However, alanine mutant is much less susceptible for quenching by FA, probably due to the weaker binding of FA than in the tyrosine mutant. Moreover, the same situation is observed with FA, where quenching ratio between minimum of PNPF159Y/FA and PNPF159A/FA differs considerably. It means that with tyrosine mutant not only FA associates better but due to the presence of additional hydroxyl group is firmly quenched.

Worth noting is the position of enzyme fluorescence minima. In Fig. 6a, it is 309 nm, which proves that quenching concerns also the tyrosinate anion fluorescence. On the other hand, in alanine mutant minimum is located at 302 nm, which indicate lack of the tyrosinate anion, and probable quenching of Tyr160 fluorescence.

**Fig. 7:**
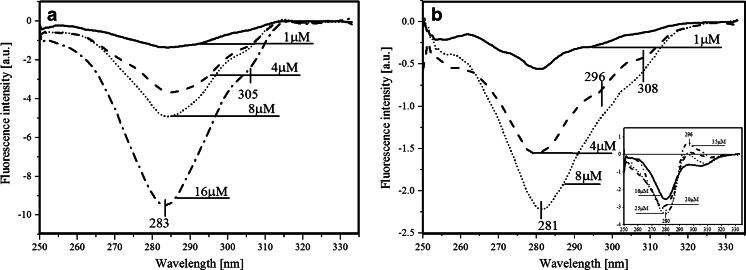
Fluorescence-excitation difference (λ_em_ 340 nm) spectra after subtraction of different FA concentrations and PNPF159Y **a** or PNPF159A **b** in 50mM Hepes pH 7.

Fluorescence-excitation difference spectra after subtraction of both components (Fig. 7a and b) also confirm quenching of the enzyme and ligand fluorescence. Nevertheless, slight shift of the excitation minima, from 277 to 281 nm (Fig. 7b) and 283 nm (Fig. 7a), may be attributed to the effect of nearby excitation band of FA fluorescence (λ_ex_ 295 nm). However, interesting observation is phenomenon with PNPF159A mutant with high FA concentrations (Fig. 7b insert). We observe there a probable FRET, the quantity of which is quite small but noticeable. According to the previous assumptions, we can only see energy transfer at high concentrations of FA, in line with its quite high K_d_. value. It seems that only concentrations high enough to create numerous complexes can exert FRET, although quite small. The reason of low quantity of FRET is probably lack of phenyl ring at 159 position.

Smaller extent of FA quenching in the complex with the PNPF159A mutant than in the PNPF159Y mutant might indicate important role of the 159 phenyl ring in π-stacking interactions. This leads to the conclusion that FRET between Tyr160 and FA does not have to be direct but might occur via the phenyl ring of phenylalanine 159 in the wild type enzyme, see also [1]. On the other hand, the addition of the hydroxyl group might incorporate changes in the π- π stacking, spatial arrangement of the aminoacid residues and ligand or might cause an orientation effect in the aspect of alignment between transition moments of mutant and FA. In addition, angle of 90° can apparently cause total decay of energy transfer probability [19], and thus eliminating FRET when compared to the wild type PNP. On the contrary, the other possible scenario could be that the RET due quenching might be performed via polypeptide chain and ended by energy dissipation.

**Fig. 8:**
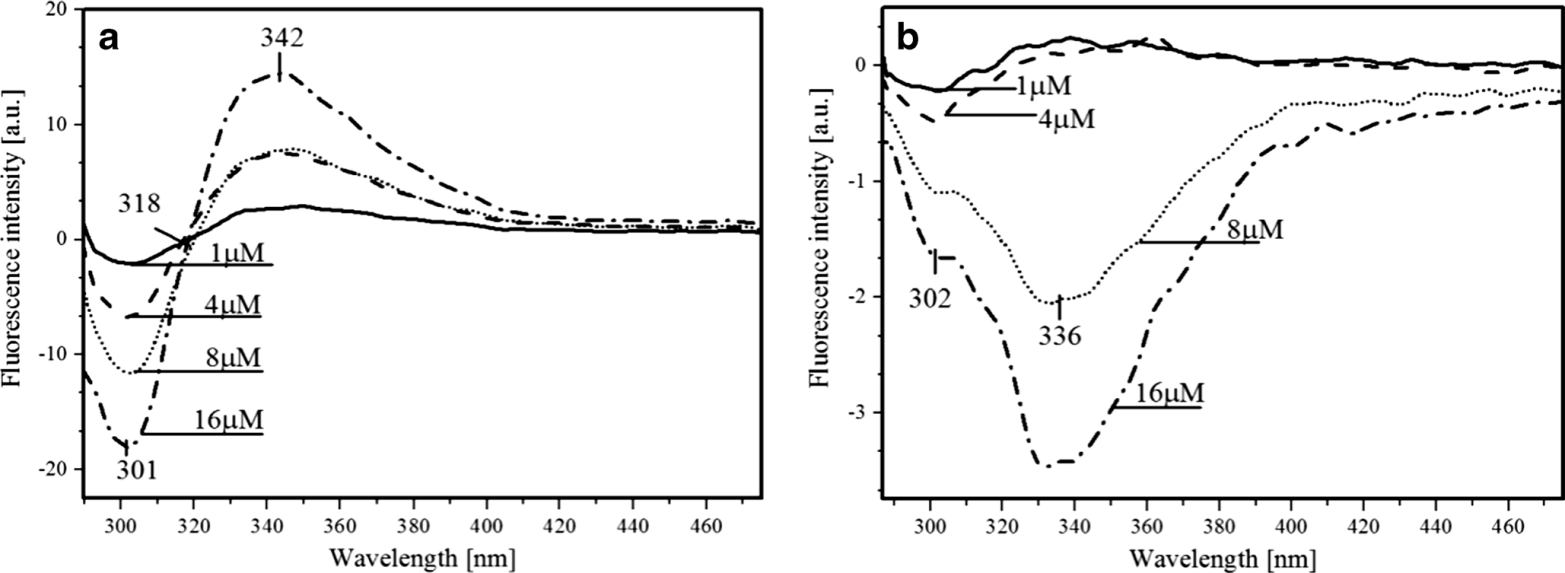
Fluorescence emission difference (λ_ex_ 280 nm) spectra of PNPF159Y **a** and PNPF159A **b** in 50mM acetate buffer, pH 5 after subtraction of the fluorescence emission spectra of FA in different concentrations and PNPF159Y **a** or PNPF159A **b** fluorescence emission spectra

Moreover the lack of FRET and presence of the tyrosinate anion are explained by the low pK_a_ value of tyrosine in S_1_ - 4.2 (pK_a_ of the ground state tyrosine is 10.6), that is caused mostly by excitation and additionally by replacement of the Phe159 by the tyrosine residue. The latter destabilizes the active site environment and facilitates tyrosine deprotonation. Hence, in pH 7 tyrosine deprotonates to the tyrosinate anion, which has red-shifted emission spectrum where formycin almost does not absorb, and probably also causes enhanced thermal energy dissipation. This statement is supported by our studies of the mutants and wild type PNP in acetate buffer in pH 5, where FRET is observable in the PNPF169Y and not in the PNPF159A (Figs. 8a and b, respectively). The latter can be explained as the result of either lower affinity of this mutant to FA (high K_d_ value) than in pH 7, or the lack of FRET — both probably caused by the absence of the phenyl ring of Phe159.

### Quantum computations of atomic charges and electronic transition dipole moments

In order to carry out the molecular dynamics (MD) simulations, it was necessary to design parameters describing interactions between the enzyme molecule and FA in both the ground and excited states of FA and Tyr160. For this purpose, quantum-mechanical SCF-CI calculations of charge distributions in selected model systems mentioned in *Methods* have been carried out to estimate changes of atomic charges with respect to the reference charges present in the standard CHARMM force fields, which were then modified by providing charges in excited states, and used in MD simulations.

**Fig. 9:**
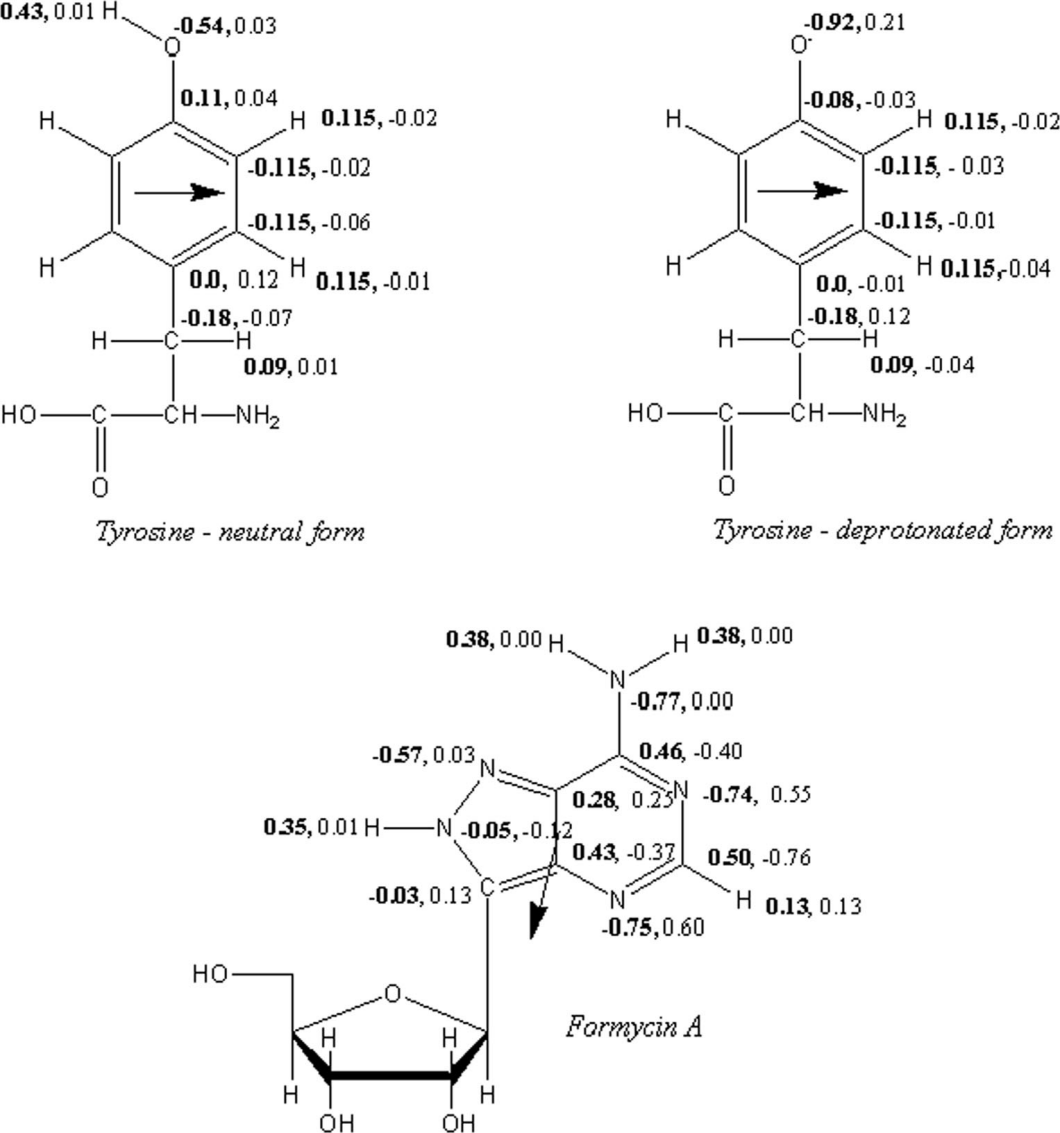
Structures, charges, and electric transition moments of tyrosine, its deprotonated form and formycin A. CHARMM charges in the ground state are in bold. Changes of the ESP charges after excitation from S_o_ to S_1_ are on the right site of the CHARMM charges. *Arrows* denote electric transition moments between S_o_ and S_1_, computed using the SCF-CI approximation with TURBOMOLE

The quantum-mechanical calculations involved the neutral molecules of Tyr, FA, and Ade, models of which have been generated using geometries from appropriate crystal structures. In particular, a model of the molecule of FA has been derived based on the crystal structure of the complex between the *E. coli* PNP enzyme, formycin B, and the phosphate anion (PDB ID: 1PR1) [20]. The carbonyl oxygen on the C6 atom in the pyrimidine ring of formycin B has been replaced with the -NH_2_ moiety, yielding formycin A. The geometry of the resultant molecule was initially optimized and then used in the QM calculations. Overall, the QM calculations allowed one to obtain ground-state point charges for all mentioned molecules and single-excitation-state point charges for Tyr and FA residues. The single-point calculations using the SCF-CI method and def-TZVP basis set [21] for all atoms have been carried out with *dscf* and *egrad* programs from the Turbomole 6.1 QM package [22]. The ESP partial charges [23] have been obtained by a fit to electrostatic potentials around the molecules. Additionally, electronic transition dipole moments (ETDM) for single-excitation transitions in Tyr and FA have also been computed with the same method. Atomic charges and ETDM are shown in Fig. 9 (below).

### Molecular dynamics simulations and FRET analysis

**Fig. 10:**
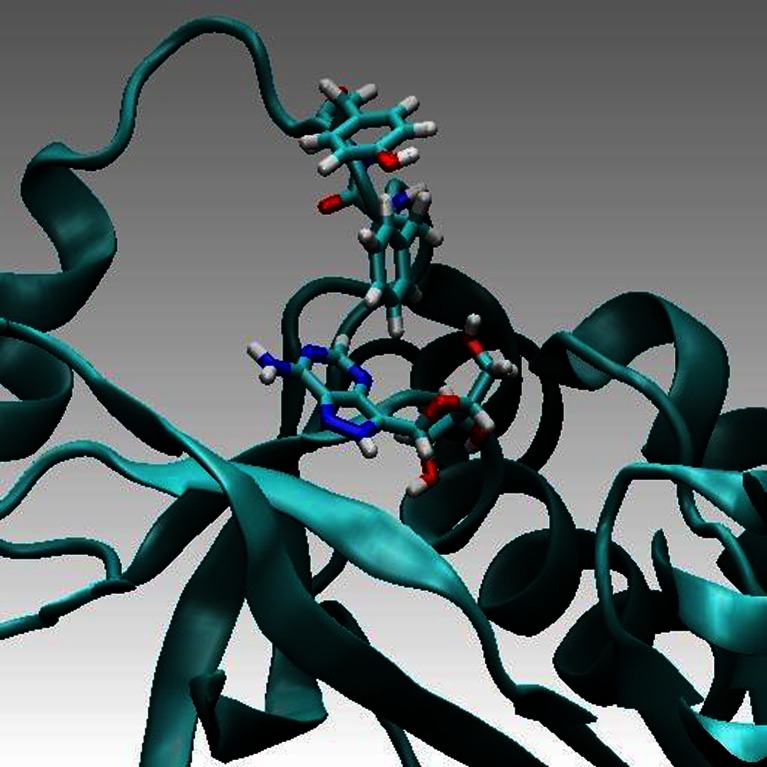
*E. coli* PNP wild-type. Phe 159 stabilizes position of FA in the binding site. For such configurations FRET is very effective

**Fig. 11:**
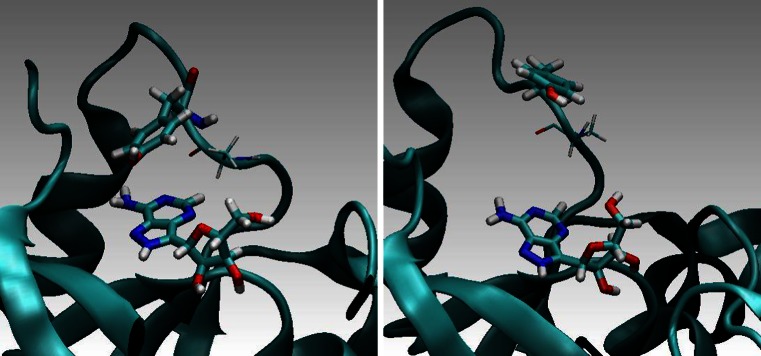
The F159A mutant of *E. coli* PNP. The lack of the phenyl ring in the 159 position results in a weaker binding and higher mobility of the ligand. FA from time to time partially “flows-out” of the binding site which results in decreasing of FRET. Above are two characteristic configurations (left — optimal for FRET, right — not optimal one)

Five ns MD simulations were carried out for complexes of the wild-type PNP and its above-mentioned mutants with FA. During this time-interval we did not observe any noticeable drift of the computed mean values (see standard error of the mean in Table 2). This indicates that the simulations were sufficiently long. MD simulations showed much better stability of the PNP (wild type) — FA complex in comparison to the PNP F159A mutant complex. In Figs. 10 and 11 (below) a few representative structural conformations in the area of the FA binding site are presented.

**Table 2:** FRET parameters calculated with the aid of QM computations and MD simulations, see Eqs. 1–8

System	<*R*_*da*_>	<*κ* ^2^>	(*κ* ^2^)_max_	<*W*_*F*_>	<*E*>
PNP, Tyr160*	9.14 ± 0.02	0.558 ± 0.004	2.23	198.3 ± 2.8	0.938 ± 0.002
PNP F159A, Tyr160*	6.50 ± 0.02	0.665 ± 0.004	1.81	2985.1 ± 40.8	0.985 ± 0.001
PNP F159Y, Tyr160*	9.35 ± 0.01	0.809 ± 0.005	2.19	177.9 ± 1.7	0.977 ± 0.001
PNP, Tyr160^(−)^*	9.24 ± 0.01	0.754 ± 0.006	3.36	~0	~0
PNP F159A, Tyr160^(−)^*	9.39 ± 0.01	0.507 ± 0.004	1.95	~0	~0
PNP F159Y, Tyr160^(−)^*	11.90 ± 0.02	1.165 ± 0.006	3.34	~0	~0
PNP F159Y^(−)^, Tyr160^(−)^*	10.29 ± 0.01	0.820 ± 0.003	1.68	~0	~0

<*R*_da_ > is the mean distance between the donor (Tyr160* or Tyr160 (−)*) and the acceptor (FA), in [Å]. <*k* ^2^ > and (*k* ^2^)_max_, are averaged and maximal orientation factors, respectively. <*W*_*F*_ > is the mean frequency of FRETs per ns. <*E* > is averaged quantum yield of the energy transfer. Standard error of the mean is also given. Zero values of < *E* > result from the zero spectral overlap integral *J* between Tyr^(−)^* as D* and FA as A, or alternatively from *R*_*F*_ = 0 (Eqs. 1–2 and 7–8)

**Fig. 12:**
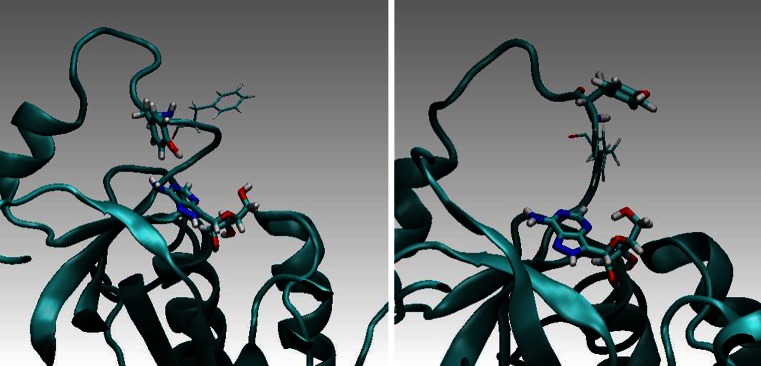
Wild-type PNP from *E. coli* in complex with FA. Characteristic are one of the most effective in FRET configurations (*left*) and an inhibiting one (*right*)

Based on MD simulations, energy transfer probabilities (ETP) were computed based on the non-radiative dipole–dipole coupling model (Eqs. 1–5). This model results in the inverse six-power dependence of the donor-acceptor distance on ETP (Eq. 1–3). The orientation factor κ^2^ of D and A was also computed and included in the analysis (Eq. 4). Total FRET probabilities were obtained by computing the FRET time-averages over the MD trajectories (Eqs. 5–8). Based on Eq. 1, the most effective and least effective configuration in FRET could be indicated and analyzed. Figure 12 (below) presents a characteristic effective and non-effective (inhibiting) configurations, left and right, respectively.

FRET efficiencies can be inferred from the photobleaching rates of the donor. Based on the above described formalism and MD simulations most important FRET parameters listed in “Theoretical methods and simulations” were calculated. They are collected in Table 2.

**Fig. 13:**
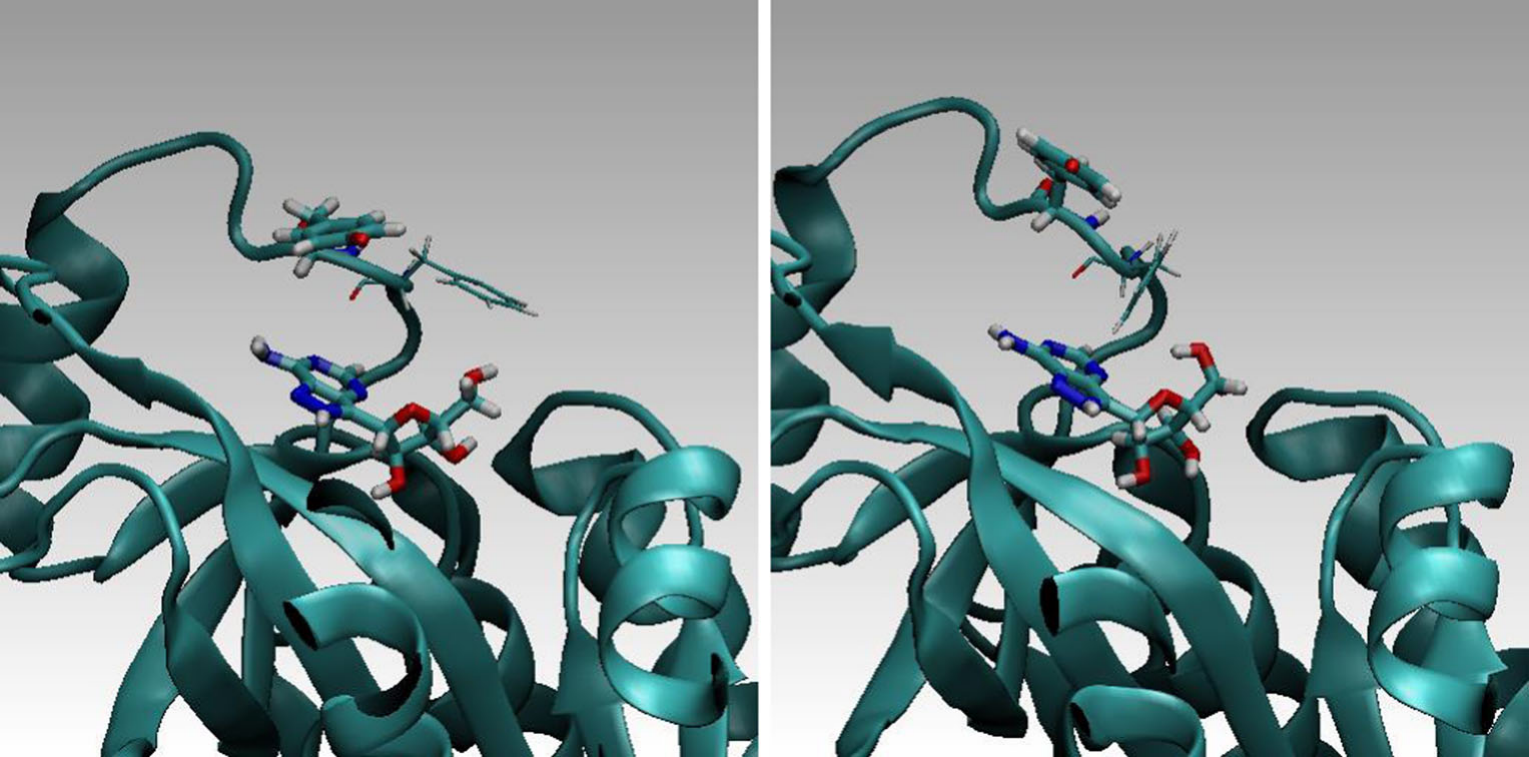
Representative snapshots from the MD simulations for the PNPF159Y mutant with Tyr160^(-)^* and in complex with FA. Characteristics are one of the most effective in FRET configurations (*left*) and an inhibiting one (*right*)

Obviously, the theoretical estimates for PNPF159A and PNPF159Y did not agree with experimental data. Based on the discussion above, we came to the conclusion that Tyr160* in these two mutants cannot exist in the neutral form. Deprotonation of this residue in its excited state explained the obvious discrepancies. The FRET parameters calculated for the following three cases, presented in Table 2, support this idea. In the case of Tyr (−)* the spectral overlap integral *J* is close to zero (hence *R*_F_ ~ 0) which makes the quantum yield of the energy transfer close to zero. Regardless of this, deprotonated Tyr160 ^(−)^* strongly interacts with the water environment leading most likely to the thermal deactivation of the tyrosinate anion. Figure 13 (below) shows a representative snapshot from the MD simulations for the PNP F159Y mutant in the complex with FA which shows that Tyr160 (−)* is directed to the solvent and Tyr159 stabilizes location of FA.

## Conclusions

**Fig. 14:**
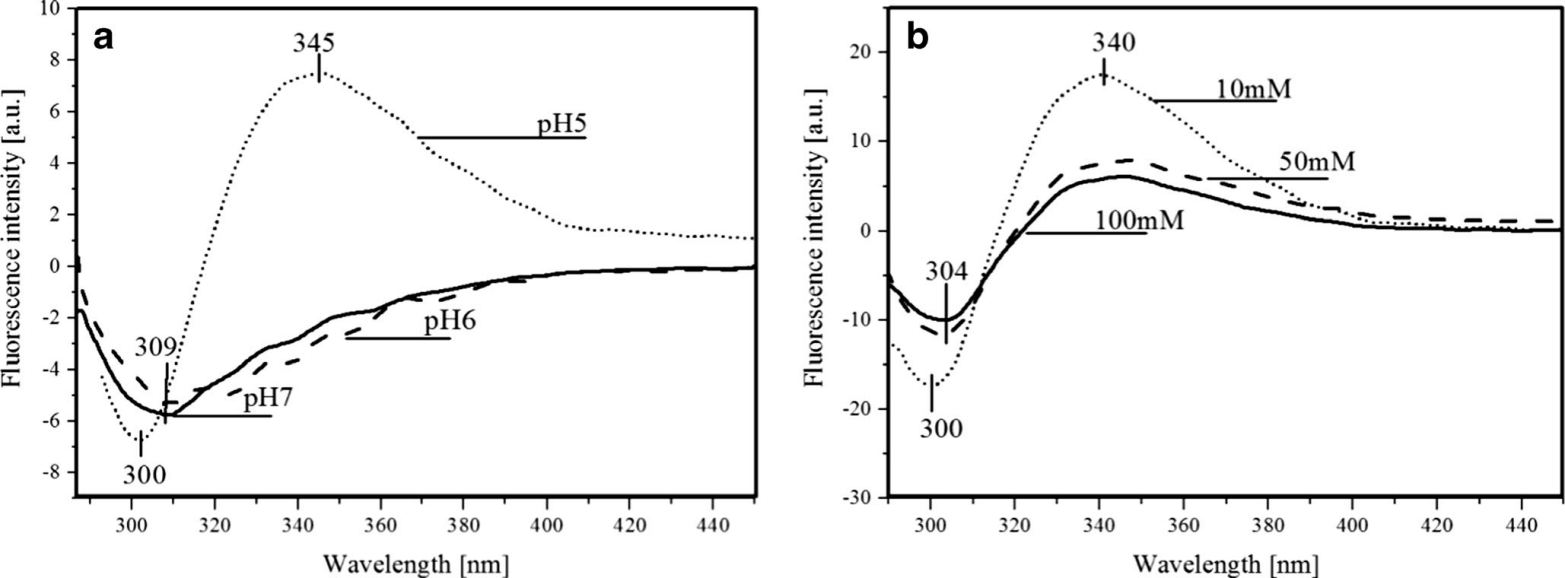
Fluorescence-emission difference spectra of 3.9 μM PNPF159Y mutant with 12 μM FA, relative to the arithmetic sum of the two components: with **a** decreasing pH values from pH 7 (Hepes buffer) to pH 6 and pH 5 (acetate buffer), and **b** decreasing concentrations of acetate buffer (pH 5)

The existence of FRET between Tyr160* and FA in the wild type PNP from *E. coli* and the lack of this phenomenon in the studied PNP mutants could not be easily explained. There were no obvious reasons why this phenomenon disappears in the mutants. In particular, when assuming existence of Tyr160* in its neutral state detailed theoretical simulations indicated existence of the FRET phenomenon. This lead us to the conclusion that in the mutants Tyr160* undergoes transition to Tyr160 ^(−)^*, drastically changing the parameters defining FRET probabilities — compare results in Table 2. The tyrosinate anion exhibits fluorescence emission spectra red-shifted (*ca*. 340 nm) when compared to neutral tyrosine (*ca*. 305 nm). The lack of FRET is explained as the result of the low pK_a_ (4,2) of the excited tyrosine in S_1_, hence at pH 7 and above it is deprotonated, which also causes enhanced thermal energy dissipation. This statement is supported by our studies of the mutants and wild type PNP in an acetate buffer at pH 5 where FRET is observable in PNPF169Y but not in PNPF159A (Fig. 8a and b). The latter can be explained as the result of either lower affinity of this mutant to FA (high K_d_ value) or lack of FRET — both probably caused by the absence of the phenyl ring of Phe159. We have also observed that the quantum yield of FRET increases due to a decrease in pH from pH 7 to pH 5 (Fig. 14a) concomitantly with a higher population of the protonated tyrosine residues in their excited state. Additionally, the PNPF159Y fluorescence emission minimum is blue-shifted to 300 nm in pH 5 (Fig. 14a) which indicates both the presence of neutral tyrosine in position 159 and the positive effect of the FA emission band. On the other hand, an increasing concentration of the common proton acceptors like acetate or phosphate (present in the buffers) leads to weaker FRET (Fig. 14b), most probably due to an increasing concentration of the anionic form of tyrosine residues.

The red-shift of the tyrosinate anion emission and thus lack of, or very slight, spectral overlap integral and thermal energy dissipation are the reasons for the FRET absence in the studied mutants at pH 7 and above. The presence of the tyrosinate anion results in a competitive energy dissipation channel and red-shifted emission, thus in consequence in the absence of FRET. These studies also indicate an important role of the phenyl ring of Phe159 for FRET in the wild-type PNP, which does not exist in the Ala159 mutant, and for the effective association of PNP with FA.

In a more general context, our observations point out very interesting and biologically important properties of the tyrosine residue in its excited state, which may undergo spontaneous deprotonation in the biomolecular systems, resulting further in unexpected physical and/or biological phenomena. Until now, this observation has not been widely discussed in the literature.

## Abbreviations

Ado: Adenosine
CI: Configurational interaction
DTT: Dithiotreitol
EDTA: Ethylenediaminetetraacetic acid
ESP charges: Electrostatic potential charges
ETDM: Electronic transition dipole moments
ETP: Energy transfer probabilities
FA: Formycin A, 3-(β-D-ribofuranosyl)-7-aminopyrazolo [4,3-d] pyrimidine
FB: Formycin B
FPLC: Fast protein liquid chromatography
FRET: Fluorescence resonance energy transfer or Förester resonance energy transfer
Guo: Guanosine
Hepes: *N*-2-hydroxyethylpiperazine-N’-2-ethanesulfonic acid
Ino: Inosine
m^7^Guo: N (7)-methylguanosine
MD: Molecular dynamics
MM: Molecular mechanics
P_i_: Orthophosphate
PMSF: Phenylmethanesulfonyl fluoride
PNP: Purine nucleoside phosphorylase
PNP: *Escherichia coli* hexameric PNP, the product of the *deoD* gene
SA: Ammonium sulfate
SCF: Self consistent field
SDS-PAGE: Sodium dodecyl sulfate polyacrylamide gel electrophoresis
TCSPC: Time-correlated single-photon counting
Tris–HCl: Tris (hydroxymethyl) aminomethane hydrochloride
TZVP: Split valence, triple zeta basis set
Xao: Xanthosine

## References

[1] Kierdaszuk B, Modrak-Wójcik A, Wierzchowski J, Shugar D (2000). Formycin A and its N-methyl analogues, specific inhibitor of *E. coli* Purine nucleoside phosphorylase (PNP): induced tautomeric shift on binding to enzyme, and enzyme-ligand fluorescence resonance energy transfer. Biochim Biophys Acta.

[2] Kierdaszuk B, Modrak-Wójcik A, Shugar D (1997). Binding of phosphate and sulfate anions by purine nucleoside phosphorylase from *E. coli*: ligand dependent quenching of enzyme intrinsic fluorescence. Biophys Chem.

[3] Hirshfield MS, Chaffe S, Koro-Johnson L, Mary A, Smith AA, Short SA (1991). Use of site-directed mutagenesis to enhance the epitope-shielding effect of covalent modification of proteins with polyethylene glycol. Proc Natl Acad Sci U S A.

[4] Kalckar HM (1947). The enzymatic synthesis of purine-ribosides. J Biol Chem.

[5] Stoeckler JD, Agarwal RP, Agarwal KC, Parks RE (1978). Purine nucleoside phosphorylase from human erythrocytes. Methods Enzymol.

[6] Kulikowska E, Bzowska A, Wierzchowski J, Shugar D (1986). Properties of two unusual and fluorescent substrates of purine nucleoside phosphorylase: 7-methylguanosine and 7-methylinosine. Biochim Biophys Acta.

[7] Błachut-Okrasińska E, Lesyng B, Briggs JM, McCammon JA, Antosiewicz JM (1999). Poisson-Boltzmann model studies of molecular electrostatic properties of the cAMP-dependent protein kinase. Eur Biophys J.

[8] Antosiewicz J, Błachut-Okrasinska, E, Grycuk T., Lesyng B (2000) A correlation between protonation equilibria in biomolecular systems and their shapes: studies using a poisson-boltzmann model. In: Mathematical sciences and applications, GAKUTO International Series. Gakkotosho Co, Tokyo. 14:11–17

[9] Hoefling M, Lima N, Haenni D, Seidel CAM, Schuler B, Grubmüller H (2011). Structural heterogeneity and quantitative FRET efficiency distributions of polyprolines through a hybrid atomistic simulation and Monte Carlo approach. PLoS ONE.

[10] Hoefling M, Grubmüller H (2013). In silico FRET from simulated dye dynamics. Comput Phys Commun.

[11] Ross JBA, Laws WR, Rousslang KW, Wyssbrod HR (1992) In: Lakowicz JR (ed.), Topics in fluorescence spectroscopy. Biochemical applications. Plenum, New York. 3:pp 1–63

[12] Mao C, Cook WJ, Zhou M, Koszalka G, Krenitsky TA, Ealick SE (1997). The crystal structure of *E. coli* purine nucleoside phosphorylase: a comparison with human enzyme reveals a conserved topology. Sructure.

[13] Włodarczyk J, Stoychev Galitonov G, Kierdaszuk B (2004). Identification of the tautomeric form of formycin A in its complex with *Escherichia coli* purine nucleoside phosphorylase based on the effect of enzyme-ligand binding on fluorescence and phosphorescence. Eur Biophys J.

[14] Kierdaszuk B (2013). Fluorescence anisotropy of tyrosinate anion using one-, two- and three-photon excitation. J Fluoresc.

[15] Bzowska A, Kulikowska E, Shugar D (1990). Properties of purine nucleoside phosphorylase (PNP) of mammalian and bacterial origin. Z Naturforsch C.

[16] Bzowska A, Kulikowska E, Shugar D (1992). Formycins A and B and some analogues: selective inhibitors of bacterial (*Escherichia coli*) purine nucleoside phosphorylase. Biochim Biophys Acta.

[17] Wu P, Brand L (1994). Resonance energy transfer: methods and applications. Anal Biochem.

[18] Koellner G, Luic M, Shugar D, Saenger W, Bzowska A (1998). Crystal structure of the ternary complex of *E. coli* purine nucleoside phosphorylase with formycin B, a structural analogue of the substrate inosine, and phosphate(sulphate) at 2.1 A resolution. J Mol Biol.

[19] Lakowicz JR (2006). Principles of fluorescence spectroscopy.

[20] Bennett EM, Li C, Allan PW, Parker WB, Ealick SE (2003). Structural basis for substrate specificity of Escherichia coli purine nucleoside phosphorylase. J Biol Chem.

[21] Eichkorn K, Weigend F, Treutler O, Alhrichs L (1997). Auxiliary basis sets for main row atoms and transition metals and their use to approximate Coulomb potentials. Theor Chem Accounts.

[22] Alhrichs R, Baer M, Haeser M, Horn H, Koelmel C (1989). Electronic structure calculations on workstation computers: the program system TURBOMOLE. Chem Phys Lett.

[23] Francl MM, Chirlian LE (2000). The pluses and minuses of mapping atomic charges to electrostatic potentials. Rev Comput Chem.

